# Empowerment, intimate partner violence and skilled birth attendance among women in rural Uganda

**DOI:** 10.1186/s12978-016-0167-3

**Published:** 2016-05-04

**Authors:** Betty Kwagala, Olivia Nankinga, Stephen Ojiambo Wandera, Patricia Ndugga, Allen Kabagenyi

**Affiliations:** Department of Population Studies, School of Statistics and Planning, College of Business and Management Sciences, Makerere University, Kampala, Uganda

**Keywords:** Empowerment, Intimate Partner Violence, Skilled Birth Attendance, Rural, Africa

## Abstract

**Background:**

There is limited research on how the empowerment of women and intimate partner violence (IPV) are associated with skilled birth attendance (SBA) among rural women in Uganda. Therefore, the aim of this paper was to investigate the association between women’s empowerment, their experience of IPV and SBA in rural Uganda.

**Methods:**

Using data from the Uganda Demographic and Health Survey (UDHS), we selected 857 rural women who were in union, had given birth in the last 5 years preceding the survey and were selected for the domestic violence (DV) module. Frequency distributions were used to describe the background characteristics of the women and their partners. Pearson’s chi-squared (*χ*^2^) tests were used to investigate the associations between SBA and women’s empowerment; and partners’ and women’s socio-demographic factors including sexual violence. Multivariable logistic regression analyses were used to examine the association between SBA and explanatory variables.

**Results:**

More than half (55 %) of the women delivered under the supervision of skilled birth attendant. Women’s empowerment with respect to participation in household decision-making, property (land and house) (co)ownership, IPV, and sexual empowerment did not positively predict SBA among rural women in Uganda. Key predictors of SBA were household wealth status, partners’ education, ANC attendance and parity.

**Conclusions:**

For enhancement of SBA in rural areas, there is a need to encourage a more comprehensive ANC attendance irrespective of number of children a woman has; and design interventions to enhance household wealth and promote men’s education.

## Background

Nearly all (99 %) maternal deaths occur in developing countries. More than half of these deaths occur in sub-Saharan Africa [1]. Although the majority of deaths are preventable, the level of decline of maternal deaths globally remains slow (at 2.6 % per year) between 1990 and 2013, short of the 5.5 % target of the Millennium Development Goal (MDG) number five [1–3]. Uganda is among the countries that did not meet the fifth millennium development goal. Her maternal mortality ratio is still high at 438 deaths per 100,000 live births, while perinatal mortality ratio stands at 40 deaths per 1000 live births [4].

Skilled birth attendance (SBA) is one of the central elements for improving maternal and newborn health [5] and a key progress indicator of the MDG 5 [6, 7]. Skilled care refers to the care provided to a woman and her newborn during pregnancy, childbirth and immediately after birth by accredited and competent health care providers, who have at their disposal the necessary equipment and the support of a functioning health system, including transport and referral facilities for emergency obstetric care. Skilled birth attendants include doctors, clinical officer/medical assistants, nurses, or midwives [6].

Utilization of skilled maternal health services in Uganda is still low. The recommended number of times of antenatal care (ANC) attendance (four times or more) was only 48 % in 2011. Skilled attendants only assisted 58 % of the births [4]. Although this was an improvement from 41 % in 2006, SBA is lower than the 63 % estimate for developing countries in general [3, 4, 8, 9]. Risks associated with unskilled birth attendance include inability to manage complications, unhygienic conditions and the use of unsterilized instruments [9, 10].

Skilled birth attendance is influenced by women’s empowerment, experience of Intimate Partner Violence (IPV), health system factors and individual or household factors. Health system factors include quality of services especially in the public health sector [1, 11–13]. Public health facilities are often characterized by inadequate equipment, low drug supply, under-staffing, poor staff motivation and cultural insensitivity [12–16]. In limited resource settings especially rural areas, limited awareness on the part of potential service users, long distances, transport challenges to health facilities and lack of waiting rooms, limit facility based delivery [13, 15, 17–19]. Evidence from sub-Saharan Africa reveals that women living in close proximity to health facilities (within five kilometres) are more likely to deliver at health facilities [1, 20, 21].

Women’s empowerment is usually associated with better health outcomes. Women’s empowerment includes household decision making, control over economic resources, and sexual empowerment. Inability to access health facilities and delays in making decisions to seek care with respect to place of delivery, has a bearing on skilled birth attendance [22]. Gender relations at household level are instrumental in determining birth attendance. In patriarchal settings, male heads play a significant role in health care associated decision-making. Women’s autonomy, social standing and feelings of independence were positively associated with skilled birth attendance in Nigeria [23] and Uganda [24]. Several studies established that women’s empowerment was not only significantly associated with modern contraception, but also skilled birth attendance [13, 25, 26]. In Busia-Uganda, involvement of other people such as a spouse in making decisions regarding place of delivery had a positive association with skilled birth attendance [27].

Sexual empowerment or what is referred to as power in sexual relationships directly or indirectly affects sexual and reproductive health [28]. Although sexual empowerment with respect to a woman’s ability to ask a partner to use a condom and to refuse sex predicted STIs in Uganda, the relationship was contrary to what was expected [29].

Intimate Partner Violence (IPV), an indicator of disempowerment of women, is a serious global public health problem with grave reproductive health consequences [30–35]. IPV has been associated with partner’s controlling behaviors including limitation of mobility, interaction with friends and family, and possibly negligent spouses who get drunk often [36, 37] possibly posing challenges for SBA. In Kenya too, lifetime experience of physical violence reduced the odds of skilled birth attendance by 29 % [38]. Similarly, IPV limited utilization of ANC and skilled birth attendance in Bangladesh [39].

With respect to individual factors, wealth or socio-economic status (SES) enhances affordability of services. Household’s wealth and a woman’s SES are associated with SBA [19, 21, 23, 27, 40, 41] in urban settlements in Kenya [13] and war affected northern Uganda [24].

Cultural factors, ethnicity and geographical locations have a bearing on the choice of birth attendance [12, 16, 19]. Adherence to traditional birth practices influences birth attendance in Uganda. While the Banyankole and Sabiny of Uganda regard childbirth as a normal process that should take place in a home setting, the Banyoro and Baganda, view it as a risky experience. Home births are valued in Uganda [12], Burkina Faso [20] and in Nigeria [23]. Despite their frequent lack of technical skills, requisite equipment and conducive environment for delivery, many ethnic groups in Uganda appreciate traditional birth attendants (TBAs) owing to their cultural sensitivity and accessibility. Traditional practices such as the use of herbs, non-supine delivery positions, placenta disposal, female genital mutilation, still influence birth attendance [12, 42, 43].

SBA is associated with urban residence [21, 23, 40, 41, 44, 45], female household headship [41] and appropriate antenatal care (ANC) attendance involving four or more times [20, 21, 44–46]. ANC attendance is an avenue for acquiring information on the status of the pregnancy. The likelihood of SBA reduces with increase in parity, especially where complications are not anticipated [17, 21, 24, 44, 46]. In Busia district of Eastern Uganda, the odds of skilled birth attendance were higher among women with parity of less than four [27]. Skilled birth attendance is usually sought for the first birth order [23].

Younger women are more likely to have SBA than older women, who could have had multiple births [23]. Religion in some cases influences reproductive health choices. In Nigeria, being Muslim reduced the odds of skilled birth attendance [23]. However, for Busia- Uganda, religion did not predict skilled birth attendance [27].

Secondary or higher level of education is associated with increase in knowledge, skills, economic empowerment and exposure, which facilitates better informed choices with respect to reproductive health. In Nigeria, education was the only individual-level factor that consistently predicted health service utilization [40]. Similar findings have been observed elsewhere in SSA [23, 41, 44–46].

The partner’s level of education is also important. Results of a study conducted in Busia—Uganda revealed that the likelihood of skilled birth attendance or delivery at a health facility was higher among household heads (93 % were male) who had secondary or higher level of education [47, 48]. However, Bbaale and Guloba [49] found that whereas both maternal and partner’s education were significant predictors, maternal education had a stronger association [50].

However, there is limited research on how empowerment of women, intimate partner violence are associated with skilled birth attendance among rural women in Uganda. Therefore, the aim of this paper was to investigate the association between women’s empowerment, their experience of intimate partner violence and skilled birth attendance in rural Uganda.

## Methods

### Data source

The study used data from the 2011 Uganda Demographic and Health Survey (UDHS). The UDHS data were accessed with permission from Measure DHS [51]. The UDHS was a cross-sectional survey that used stratified two-stage cluster sampling design [4], which was used in the 2002 population and housing census [52]. Detailed description of sampling procedures is reported in the UDHS report [4].

The 2011 UDHS interviewed 8674 women age 15–49 years. We selected rural women who were in union, had given birth in the last 5 years preceding the survey and were selected for the domestic violence (DV) module. This resulted into a weighted sample 857 women. The DV module was based on the shortened and modified version of the Conflict Tactics Scale [53]. The survey was carried out based on World Health Organization’s (WHO) ethical and safety recommendations for research on domestic violence [54].

### Measure of outcome variable

The outcome variable “skilled birth attendance” (SBA) was measured using the question: who provided assistance during delivery for the most recent birth? This question was asked to women who had had a live birth in the last five years preceding the survey. Respondents who were assisted by a skilled provider (doctor, nurse/midwife and medical assistant/clinical officer) were recoded as having a skilled birth attendant and all others were recoded otherwise. It is important to note that we considered data on the most recent birth by the women. Thus the outcome variable SBA was recoded as a binary outcome.

### Measures of women’s empowerment

Four indices were created to measure empowerment of women: decision making, economic empowerment, access to healthcare and sexual empowerment indices. In addition, IPV was used as an indicator of disempowerment of women. Decision making included women’s responses to questions on who decides on (1) their own health; (2) large household purchases; and (3) visits to their family and (4) control over their earnings. Responses to these questions were re-coded into two categories (1 = woman decides alone/jointly with partner, 0 = partner alone/others). Women’s participation in decision-making included their individual or joint participation (with their partners) in any of the four questions.

Economic empowerment included women’s responses to questions on: whether the woman owns a house; owns land; and the type of earning from her work. Women who owned a house or land (either alone or jointly with a partner) or received cash payment for their work, were recoded into economically empowered women and all others were recoded as not being economically empowered (1 = Yes, 0 = No).

Sexual empowerment was measured using two questions: ability to refuse sex or ask a partner to use condoms. Sexually empowered women were those who were able to refuse sex and ask their partners to use condoms during intercourse. This was recoded as binary variable (1 = sexually empowered; 0 = Not sexually empowered).

We developed an index for healthcare access challenges for the women using two questions: whether a) getting money needed for treatment and b) distance to a health facility, were significant problems preventing them from seeking medical care. Women who reported that either getting money or distance were a big problem, were recoded to be having access problems. Those who reported that it was not a problem were recoded into a “no” category implying that access to healthcare was not a problem.

Experience of IPV in the last 12 months preceding the survey combined two questions: physical (combined variables d106 and d107) and sexual violence (variables d108). We added the two variables to form one aggregate measure of violence. Women who reported either physical or sexual violence were recoded into a 1 (yes) and 0 (no) for those who experienced none of the forms. The questions asked to measure intimate physical violence in the last 12 months are reported elsewhere [36]. Those for measuring intimate partner sexual violence are reported in another article [37].

### Measures of other explanatory variables

Other explanatory variables included women’s and their partners’ background factors. Women’s background factors included ANC attendance, age group, region of residence, wealth index, religion, educational level, household headship, and number of children ever born.

Number of ANC visits were captured from the responses on the question of how many times the respondent received antenatal care during pregnancy. Since WHO guidelines [55] recommend at least four visits, women who reported four or more times were grouped into having adequately attended ANC and those who had less than four visits were grouped as not having adequately attended ANC.

Number of children ever born (parity) was recoded into three categories: 1 = one child, 2 = 2–4 children, and 3 = 5+ children. Region was recoded as central, eastern, northern and western regions. Religion was recoded as Catholics, Protestants, Muslims and Pentecostals/Others. The category ‘Others’ comprised smaller religious groups, such as Seventh Day Adventists [SDAs].

Wealth status was assessed using wealth index. Wealth index is a composite measure of a household’s cumulative living standard. It is calculated using data on a household’s ownership of assets, such as televisions and bicycles; materials used for housing construction; and types of water access and sanitation facilities [56]. For this study it was recoded into four quintiles: poorest, poorer, middle, and rich (combined richer and richest due to smaller numbers in each of these categories). Women’s education level was recoded into three categories: none, primary and secondary or higher. Age was grouped into four categories: 15–19, 20–29, 30–39 and 40–49.

Partner’s characteristics included age group, educational level and drinking alcohol. Partners’ age was recoded into four categories: 15–19, 20–29, 30–39 and 40–49. Partners’ education level was recoded into three categories: none, primary and secondary or higher. Alcohol drinking was a binary variable (0 = No, 1 = Yes).

### Statistical analysis

Frequency distributions were used to describe the background characteristics of the women and their partners. Cross-tabulations were used to investigate associations between SBA (dependent variable) and women’s empowerment (decision making, economic empowerment, access to healthcare, sexual empowerment indices; IPV); and partners’ and women’s socio-demographic factors. Pearson’s chi-squared (*χ*^2^) tests were used to examine the significant differences between SBA and the explanatory variables. The level of statistical significance using p-values was set at *p* < 0.05. However, IPV (*p =* 0.085) and decision making index (*p =* 0.631) were included as a priori variables based on the literature reviewed.

Multivariable logistic regression analyses were used to examine the association between SBA and explanatory variables whose *p*-values were less than 0.05 during the chi-square tests, with the exception of some of the women’s empowerment indices including IPV. These variables were added as a priori variables due to literature reviewed. Results are presented in the form of Odds Ratios (OR) reporting 95 % confidence intervals. The level of statistical significance using *p*-values was set at *p* < 0.05. All analyses were weighted to adjust for non-response and disproportionate selection using the domestic violence weights and performed in STATA version 13.

## Results

### Descriptive characteristics

Table 1 presents the descriptive results. Table 1 shows that 55 % of rural women had skilled birth attendance. More than half (59 %) of the women (*n =* 858) were below 30 years. About 46 % of the women had delivered five children or more. Over half of the respondents were Christian; 43 % were Catholics while 28 % were Protestants. Only 18 % had secondary or higher education. Most of the households were male-headed (84 %). Over half (54 %) had less than five children. Close to half (49 %) had attended ANC four or more times.

**Table 1:** Distribution of women by their demographics, socio-economic factors, their partners’ characteristics and skilled birth attendance during the recent delivery

	% of women	Frequency	% SBA	*p*-value
Age group				0.449
15–19	6.1	53	64.2	
20–29	52.6	451	55.9	
30–39	31.5	271	53.8	
40–49	9.7	84	47.7	
Region				0.210
Central	20.9	179	63.5	
Eastern	29.4	252	57.6	
Northern	22.0	189	48.9	
Western	27.7	238	50.6	
Wealth Index				<0.001
Poorest	24.2	208	39.7	
Poorer	25.2	216	50.5	
Middle	23.2	199	54.5	
Rich	27.4	235	72.9	
Women’s Education level				<0.001
None	18.7	160	38.7	
Primary	63.5	544	53.5	
Secondary+	17.9	153	77.3	
Religion				0.540
Catholic	43.1	369	55.2	
Protestant	28.1	241	56.2	
Muslim	11.7	100	59.5	
Pentecostal and others	17.2	147	49.4	
Sex of a household head				0.370
male	84.4	724	54.2	
female	15.6	134	59.2	
Number of children ever born				<0.001
1	11.9	102	77.6	
2–4	42.2	362	55.3	
5+	45.9	394	48.9	
Attended ANC four or more times				<0.001
No	51.2	439	44.8	
Yes	48.8	418	65.7	
Participates in decision making in any of the four issues				0.630
No	15.6	134	57.9	
Yes	84.4	724	54.4	
Economically empowered - owns house or land or earns cash				0.003
No	14.1	121	70.2	
Yes	85.9	737	52.5	
Access to health facility is a big problem				0.015
No	31.5	271	62.0	
Yes	68.5	587	51.7	
Can refuse sex and ask partner to use condom				0.001
No	33.5	287	46.6	
Yes	66.5	571	59.2	
Experienced intimate partner violence				0.085
No	47.1	404	59.4	
Yes	52.9	454	51.0	
Partner’s age				0.524
16–29	30.9	265	58.6	
30–39	39.4	338	53.6	
40+	29.8	255	53.0	
Partner’s education level				<.001
None	11.1	95	33.7	
Primary	61.2	525	50.7	
Secondary+	27.7	238	72.8	
Partner drinks alcohol				0.187
No	52.4	449	58.2	
Yes	47.6	408	51.4	
Total	100.0	858	55.0	

With respect to empowerment and associated factors, 84 % of the mothers participated in decision making addressing at least one key household issue; 86 % owned either a house or land individually of jointly with their partners. Over half (69 %) had challenges in accessing health facilities and 53 % experienced IPV. The majority (67 %) could refuse sex and could ask a partner to use a condom. Seven in ten of partners were below the age of 40; 72 % had primary or no education and 48 % drunk alcohol.

Table 1 also shows the results of the cross tabulation (chi-square tests) between SBA and selected factors. SBA is not significantly associated with women’s empowerment with respect to decision-making, but is significantly associated with economic empowerment, and sexual empowerment. Access to a health facility was significantly associated with SBA but IPV had no significant association.

Among other independent factors, skilled birth attendance was significantly associated with ANC attendance, number of children ever born, wealth index, woman’s level of education, and partner’s education. SBA was higher among women who did not own a house or land (70 %), where access to a health facility was not a big problem (62 %), who could refuse sex (59 %) and did not experience IPV (59 %). SBA was also higher among women who had one child (78 %), attended ANC four or more times (66 %), from rich households (73 %), with secondary or higher level of education (77 %), and had partners with secondary or higher education (73 %). Region, religion, sex of the household head, partner’s age, and partner’s alcohol consumption where not significantly associated with SBA.

### Association between women’s empowerment, IPV and skilled birth attendance

In Table 2, we estimated multivariable logistic regression models to examine the relationship between skilled birth attendance and women’s empowerment, controlling for access to health care, ANC attendance, women’s and partner’s background characteristics. The first model consisted of the main predictor variables namely: participation in household decision making, economic empowerment, access to health facility, sexual empowerment, and intimate partner physical violence. In the second model, we added women’s background characteristics and in the third model, we added partners’ characteristics. Participation in household decision-making and IPV were included in the model although they were not significantly associated with SBA at bivariate level of analysis being key measures of women’s empowerment and because of their importance in literature.

**Table 2:** Logistic regression of skilled birth attendance for the women’s recent birth and their empowerment, socio-economic and partner’s factors (DHS 2011)

	Model (1)		Model (2)		Model (3)	
Variables	OR	95 % CI	OR	95 % CI	OR	95 % CI
Participates in decision making in any of the four issues (rc = no)	0.93	[0.49–1.76]	1.07	[0.61–1.88]	1.09	[0.62–1.92]
Economically empowered - owns house or land or earns cash (rc = no)	0.48^**^	[0.27–0.83]	0.49^*^	[0.28–0.86]	0.51^*^	[0.29–0.88]
Can refuse sex and ask partner to use condom (rc = no)	1.64^**^	[1.21–2.22]	1.28	[0.91–1.81]	1.26	[0.89–1.78]
Experienced intimate partner violence in 12 months (rc = no)	0.71	[0.47–1.08]	0.86	[0.57–1.30]	0.86	[0.57–1.29]
Access to health facility is a big problem due to distance & finances (rc = no)	0.72	[0.51–1.02]	0.98	[0.67–1.43]	0.97	[0.66–1.43]
Attended ANC four or more times (rc = no)			2.01^***^	[1.39–2.90]	1.93^***^	[1.34–2.79]
Wealth index (rc = poorest)						
Poorer			1.46	[0.89–2.41]	1.33	[0.79–2.26]
Middle			1.51	[0.87–2.60]	1.40	[0.80–2.46]
Rich			2.84^***^	[1.58–5.12]	2.59^**^	[1.41–4.76]
Women’s education level (rc = none)						
Primary			1.37	[0.86–2.19]	1.17	[0.70–1.95]
Secondary or higher			2.54^**^	[1.36–4.74]	1.86	[0.94–3.65]
Number of children ever born (rc = 1)						
2–4			0.38^**^	[0.21–0.71]	0.40^**^	[0.22–0.74]
5+			0.37^**^	[0.20–0.70]	0.40^**^	[0.21–0.75]
Partner’s education level (rc = none)						
Primary					1.52	[0.84–2.74]
Secondary or higher					2.31^*^	[1.18–4.50]
Observations	897		897		897	

^*^ *p <* 0.05- ^**^ *p <* 0.01-

^***^ *p < 0.001;* rc = reference category

*OR* Odds Ratios

*CI* confidence interval

Model 1- Considered main predictor variables

Model 2- added women’s factors

Model 3- added partners’ education

Among the main predictors, only economic empowerment consistently retained its significance after controlling for ANC attendance, partners’ and women’s background factors. Women’s ability to refuse sex and ask partner to use condom lost significance after controlling for ANC attendance, partner factors and women’s background factors. Surprisingly, compared to women that did not own property independently or jointly with their partners, women who owned property consistently had reduced odds of SBA (with a final AOR =0.51; CI 0.29–0.88). In the first model, compared to women who could not refuse sex or ask for a condom, the odds of SBA were higher among women who could refuse sex and ask partner to use condom (AOR = 1.64; CI 1.21–2.22). Participation in decision-making, access to health facility; and IPV were not significantly associated with SBA in any of the three models.

ANC attendance and number of children ever born predicted SBA. ANC of four or more times increased the odds of SBA (AOR = 1.93; CI 1.34–2.79). Women who had two or more children had reduced odds of SBA compared to women who had experienced one birth (AOR = 0.40; CI 0.22–0.74 and AOR = 0.40; CI 0.21–0.75 women who had 2–4 and 5 or more children respectively).

Wealth index and the partner’s education were significantly associated with SBA. The odds of SBA were higher for women from rich households relative to their poor counterparts (AOR = 2.59; CI 1.41–4.76). Women who had partners with secondary or higher education had increased odds of SBA compared to those whose partners had no formal education (AOR = 2.31; CI 1.18–4.50).

ANC attendance, children ever born, and wealth index remained significant in the second and third models. Women’s education was significant with increased odds of SBA for women with secondary or higher education compared to women with no formal education but lost its significance after controlling for partner’s education.

## Discussion

The prevalence of SBA among rural women (55 %) is lower than the national prevalence of 58 % and the 63 % estimate for developing countries [3]. Significant predictors of SBA were economic empowerment, ANC attendance, wealth index, number of children ever born, and partners’ education.

Women’s empowerment with respect to participation in decision making on key household issues, including their own health care, did not predict SBA. This finding is contrary to studies elsewhere which established that women’s autonomy [22] and decision making in particular in Busia—Uganda [27], was a significant determinant of SBA.

Contrary to other studies, sexual empowerment (a form of decision making) with respect to a woman’s ability to refuse sex and ask for a condom [29]; intimate partner violence [38]; and access to health services [1, 13, 17–21], did not predict SBA. Sexual empowerment was only significant in model 1. When other covariates were adjusted for, it ceased to be significantly associated with SBA. The possible explanation could be that the sample size was small since we focused on rural women selected and interviewed in the domestic violence module only.

Results on the association between women’s economic empowerment measured by ownership of land or a house (individually or jointly with her partner) and SBA reveal an opposite relationship to what was expected. Ownership of land or a house actually reduced the odds of SBA. The type of land tenure, the size of the land and the quality and size of the house matter. The UDHS shows that the quality of shelter of the majority of Ugandan is still inadequate; where eight in ten of houses had earth, sand or dung floors and over seven in ten with one to two rooms [4]. A study conducted in Nepal also confirmed that land and house size did not predict SBA [57]. Although land is an important resource, it does not easily translate into finances that could facilitate SBA.

Household wealth index, a proxy indicator of income and a more holistic measure of standard of living [58], was the strongest predictor of SBA. Although it does not address issues of gender-based control over resources, it suggests that women’s maternal health is dependent on the overall wealth status of their households. Higher odds of SBA among women from rich households relative to poor households has been observed elsewhere in the developing world [19, 21, 23, 27, 40, 41].

As observed elsewhere in SSA, adequate ANC attendance was significantly associated with SBA [20, 21, 44–46]. ANC attendance of four or more time implies appreciation of and adherence to [23, 40] the recommended maternal health care. Similar to findings elsewhere, parity predicted birth attendance with reduced odds of SBA among multiparous women [17, 21, 23, 24, 27, 44, 46].

Partner’s education was a strong predictor of SBA [47, 48]. Whereas a woman’s level of education is a key ingredient of women’s wellbeing and empowerment [49, 50, 59], in this case, after controlling for partner’s education, it did not predict SBA.

Notwithstanding the strength of this study, there were some limitations with the data. Our study was based on cross-sectional and secondary data. The dataset had no variables on type of land tenure or land size to facilitate better assessment of economic empowerment. Measurement of processes and determination of causal relationships was not easy to ascertain. Nevertheless, it points to important programmatic areas of intervention for promoting SBA in rural areas.

## Conclusions

Women’s empowerment with respect to participation in household decision-making, property (land and house) (co)ownership, IPV, and sexual empowerment did not positively predict SBA among rural women in Uganda. Key predictors of SBA were household wealth status, partners’ education, ANC attendance and parity.

For enhancement of SBA in rural areas, there is a need to encourage a more comprehensive ANC attendance irrespective of number of children a woman has; and design interventions to enhance household wealth and promote men’s education.
